# Treatment of Sleeplessness

**Published:** 1907-01

**Authors:** 


### THE TREATMENT OF SLEEPLESSNESS.

---

The *Practitioner* for July, 1906, contains an article by Broadbent on this topic. He enumerates some of the most obscure causes of sleeplessness. One is high arterial tension. High blood-pressure is not by any means invariably attended with sleeplessness, but it is a contributory cause of the sleeplessness of old age, of arterial degeneration, and of renal disease. The blood-pressure overcomes the resistance in the cerebral arterioles, and maintains an active blood supply to the cortex, which is inconsistent with sleep. When no other cause of habitual difficulty and delay in going to sleep can be found, and the pulse tension is high, the possibility that this is the cause must be entertained.

A characteristic of this form of sleeplessness is the possibility of getting off to sleep. In such cases a dose of calomel is usually the best remedy, and the patient, who has had a calomel and colocynth pill, will often ask what powerful opiate has been given. It is interesting to remark that the sleep is obtained at once, while the aperient action only takes effect in the morning. By milder mercurial aperients, every other night or twice a week, the arterial tension may be kept down, and sleep obtained indefinitely, but where high pressure in the arterial system has been identified as a cause of insomnia all the dietetic and eliminant measures, by means of which the blood-pressure is reduced, must be put in operation.

Chloral has a specially favorable hypnotic influence when the blood-pressure is high, as it relaxes the peripheral vessels, and it owes much of its credit to its good effects in these cases.

Sleeplessness due to very low blood-pressure is less common. Low pulse tension, however, is probably a factor in the sleeplessness of acute febrile diseases, though the main cause is a toxin. The effect of a cold bath, or of tepid or cold sponging, in inducing sleep in pyrexia, of whatever kind, must be due to the tonic influence on the cardivascular system, and is attended with improvement in the pulse tension.

In neurasthenia, again, the cardiovascular asthenia is a contributory cause of the sleeplessness which attends this condition.

The special feature of low tension sleeplessness is that the patient is drowsy, and will fall asleep in a chair while sitting up, but is wide-awake the moment he lies down. After a restless night he may fall fast asleep while at breakfast or soon after. The atony of the arterioles is such that they oppose no resistance to the effect of gravity; the cortical capillaries are full when the head is low, empty when it is raised. A similar effect is seen in some forms of heart disease, but produced by venous obstruction rather than by arterial asthenia. Sleep is impossible in the recumbent position, and the worn-out sufferer spends the weary night upright in his chair.

The remedy in low tension sleeplessness is not a narcotic, but a cardivascular tonic. Digitalis, or theobromine, or caffeine will be given as well as the particular tonic indicated by the general condition. But very frequently a teacup of strong, hot beef tea will send the patient to sleep at once, or even a cup of tea or coffee, which is generally antagonistic to sleep. Beef tea is better than milk, as imposing less work on the stomach, and the temperature is a matter of importance; the quantity must be small.

By far the most common cause of sleeplessness, however, is gastrointestinal derangement, especially when associated with flatulence, and while the distention of any part of the alimentary canal may give rise to dreams and broken rest, or definite wakefulness, it is gastric dyspepsia which gives nightmare, and dilatation of the stomach which produces the most serious and obstinate sleeplessness. Associated with the sleeplessness there is often a degree of depression, which is not explained by the mere loss of rest, together with irritability, and a tendency to look on the dark side of affairs. One of the first things, therefore, to be done in a case of sleeplessness is to inquire carefully and thoroughly into the state of the digestion, and to make a physical examination of the abdomen. Any derangement which may be discovered must be rectified. One form of sleeplessness is so common in association with dilatation of the stomach as almost to be characteristic. The patient goes to sleep at once on getting into bed, but in one, two, or three hours wakes up with remarkable punctuality, it may be quietly, without obvious discomfort, or with a start or bad dream, or actual nightmare, or with violent palpitation or pain in the region of the heart, or in profuse perspiration. He is at once particularly wide-awake, and the brain seems to be preternaturally active. If he has worries they crowd into his mind and are set down as the cause of his wakefulness; if he has no definite troubles, he invents or goes in search of them. The explanation of the waking up at a given time is that the stomach not having got rid of its contents, fermentation of the stagnant matters takes place, and after a certain time sufficient gas and acid are produced to put an end to sleep. Sometimes a hungry sinking feeling is experienced, and some form of nourishment is taken, after which the patient may sleep, and he attributes the waking up to exhaustion. The sleep, however, is due to the displacement of gas by the food taken, or to the dilution of acrid matters in the stomach. A dry biscuit is often more effectual than liquid nourishment.

It is noteworthy that such patients make no complaint of flatulence, although the stomach resonance may be very extensive and tympanitic, and may invade the chest up to the fifth space or rib. While we are conscious of flatulence, it is not of the presence of gas as such, but of the attempt to get rid of it, or to pass it on by peristalsis.

In such cases the diet must be simplified and the stomach must never be distended. The amount of bread and other farinaceous foods, of green vegetables, and of liquid with meals must be restricted, water hot or cold being drunk night and morning. One or two tablespoonfuls of brandy or whisky in a claret glass of hot water as the only drink at lunch and dinner will often be useful in dislodging gases.

Treatment will be directed to the prevention of fermentation in the stomach and intestinal canal, for which purpose salol, naphthol, creosote, carbolic acid, or sulphocarbolates may be given, not forgetting calomel or other mercurial, usually a very important part of the remedies.

The sleeplessness due to dilatation of the stomach is very common and the recognition is of much importance, on account of the depression which may attend it.—*Therapeutic Gazette*, Nov. 1906.

